# Managing recurrent parvovirus B19-associated anemia after a pediatric kidney transplant

**DOI:** 10.1007/s00467-024-06378-6

**Published:** 2024-05-22

**Authors:** Mehmet Sabanci, Mehmet Taşdemir, Burcu Öksüz, Yasemin Altuner Torun, Murat Sütçü, Ozan Özkaya

**Affiliations:** 1 İstinye University School of Medicine, Istanbul, Turkey; 2 Division of Pediatric Nephrology, Department of Pediatrics, İstinye University School of Medicine, Azerbaycan Caddesi, No: 4/A, Sarıyer, Istanbul, Türkiye; 3 Molecular Infection Laboratory, Genetic Diseases Diagnosis Center, İstinye University, Istanbul, Turkey; 4 Division of Pediatric Hematology, Department of Pediatrics, İstinye University School of Medicine, Istanbul, Turkey; 5 Division of Pediatric Infectious Disease, Department of Pediatrics, İstinye University School of Medicine, Istanbul, Turkey

**Keywords:** Anemia, Immunosuppression, Kidney transplant, Parvovirus B19, Treatment protocols

## Abstract

A 13-year-old girl who had a kidney transplant four weeks prior presented with a 10-day history of fatigue, paleness, and headache. On physical examination, tachycardia and paleness were noted. Laboratory testing was notable for severe anemia and mild leukopenia and thrombocytopenia. Polymerase chain reaction (PCR) test for Epstein-Barr virus (EBV) and cytomegalovirus (CMV) were negative and for parvovirus B19 (PVB19) was positive. Despite lower immunosuppression and administration of intravenous immunoglobulin (IVIG) it persisted for 15 months, and frequent red blood cell transfusions were needed. PVB19 is a less common but significant complication. The patient's clinical course demonstrates the importance of this complication and the challenges in its management. A notable void exists in the literature regarding standardized treatment protocols for PVB19-induced recurrent anemia after kidney transplant. This case indicates the need for further research and consensus to guide effective clinical interventions in similar cases.

## Case report

A 13-year-old girl presented with a 10-day history of fatigue, paleness, and headache. She had undergone a paired kidney transplant four weeks earlier due to kidney failure associated with a neurogenic bladder. The donor was a 52-year-old woman with no human leukocyte antigen (HLA) match (2A, 2B and 2DR mismatches). The patient was given a dose of 2.5 mg/kg/day rabbit anti-thymocyte globulin (rATG) as an induction therapy (due to no HLA match) at days 1, 2 and 3, and perioperatively 30 mg/kg of methylprednisolone. Maintenance immunosuppression was planned to include tacrolimus (0.15 mg/kg/day), mycophenolate mofetil (MMF, 1200 mg/m^2^/day), and steroid. The methylprednisolone dose was tapered as follows in the initial 3 weeks: daily 15 mg/kg, 10 mg/kg, 7.5 mg/kg, 5 mg/kg, 2.5 mg/kg, 2 mg/kg, 1.5 mg/kg, 1 mg/kg, and subsequently weekly 0.5 mg/kg, 0.25 mg/kg, and 0.15 mg/kg continuously.

On physical examination, her height was 143 cm (3p), weight 40 kg (26p) blood pressure 112/78 (95p: 119/79) mmHg, body temperature 36.9° C, pulse 119 beats/min, respiration rate 18 breaths/min. Skin color was pale. Tenderness, redness, or rash were absent in the transplant kidney region. Other findings were unremarkable.

Laboratory studies revealed leukocyte count was 3.88 K/uL, polymorphonuclear leukocyte count 2.82 K/uL, hemoglobin 6.7 g/dL, and platelet count 123 K/uL. Levels were: blood urea nitrogen (BUN) 11 mg/dL (N 5–18 mg/ dL), serum creatinine 1.11 mg/dL (N 0.5–1.0 mg/dL), albumin 4.1 g/dL (N 3.8–5.4 g/dL), sodium 139 mmol/L (N 136–145 mmol/L), potassium 4.1 mmol/L (N 3.5–4.5 mmol/L), serum tacrolimus 10.0 ng/mL, and CRP 0.1 mg/dL (N < 0.3 mg/dL). Urinalysis revealed absence of protein, leukocytes, erythrocytes, or glucose.

At six weeks of follow-up, the patient stated that she had headache and fatigue. She was on 5 mg prednisolone, 2 mg tacrolimus, and 1500 mg MMF. The serum tacrolimus level was between 4.4–10 ng/mL within the first month. Laboratory tests revealed hemoglobin levels reduced to 5 g/dL, necessitating immediate intervention and evaluation. Due to leukopenia and severe anemia, MMF adverse effects were considered and MMF dosage was dropped to 600 mg/m^2^/day. Peripheral blood smear was normocytic normochromic, direct Coombs test was negative, and the levels of haptoglobin, serum iron, vitamin B12, and folic acid were within normal range. She immediately was given 2 units of red blood cells to address the anemia. The anemia persisted over the next 2 weeks. A polymerase chain reaction (PCR) test was negative for Epstein-Barr virus (EBV) and cytomegalovirus (CMV) and positive for parvovirus B19 (PVB19), with over 50 million IU/mL copies. Also, parvovirus IgM was positive (1.40, N < 1.1), and parvovirus IgG was negative. As a result, tacrolimus and MMF dosages were gradually tapered. Two months later due to persistent severe anemia (hemoglobin 5.3 g/dl) and high viral load (PVB19, > 50 million IU/mL), a course of intravenous immunoglobulin (IVIG) was given (0.5 g/kg four times over a month).

**Fig. 1:**
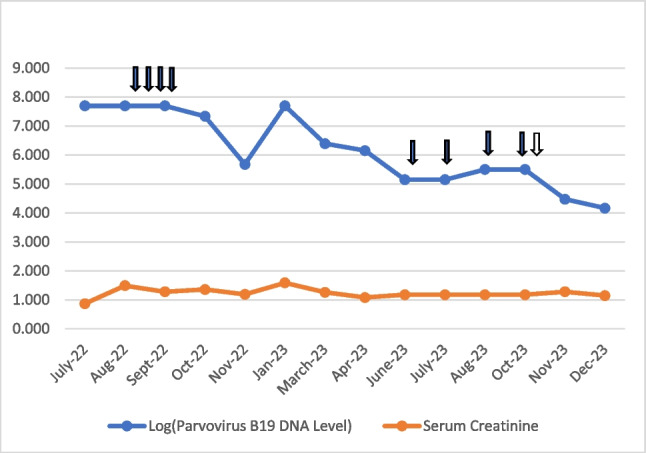
Illustration of a logarithmic graph capturing the temporal trends of log-transformed parvovirus B19 DNA levels, unaltered serum creatinine concentrations, the occurrence of intravenous immunoglobulin (IVIG) administration (denoted by black arrows), and the commencement of everolimus (indicated by a white arrow) spanning the timeline from July 2022 to December 2023. The figure provides a comprehensive visual depiction of the patient's therapeutic response, correlating variations in viral load with specific treatment milestones and immunosuppressive adjustments

During 11 months of subsequent follow-up, despite low-dose immunosuppression (prednisone, tacrolimus, and MMF) and IVIG therapy, she required 10 units of red blood cell transfusions. At 12 to 16 months after transplantation, IVIG was re-administered monthly at a dose of 0.5 g/kg (four times). Viral load declined to 140.800 IU/mL copy number but she still had severe anemia episodes and needed red blood cell transfusions five times in four months. At 16 months after the kidney transplant, MMF was switched to everolimus. After that, hemoglobin level was significantly improved from 5 g/dl to 11.9 g/dl within 2 months, without red blood cell transfusion. Figure 1 shows the changes elucidating the temporal dynamics of parvovirus B19 DNA levels, serum creatinine concentrations, the initiation of everolimus, and the instances of IVIG administration throughout the patient's therapeutic course.

## Discussion

Parvovirus B19 (PVB19) infection in immunosuppressed patients, especially kidney transplant recipients, presents a range of challenging clinical manifestations. Patients often exhibit a cluster of symptoms, including fever, rash, arthralgia, and fatigue. Anemia is a significant concern and may even develop to pure red cell aplasia (PRCA). Numerous studies report anemia in 25%–50% of kidney transplant recipients (KTRs), suggesting it is associated with immunosuppressives, hemolysis, frequent blood sampling, vitamin B12, folic acid or iron deficiency, and infections (especially, EBV, CMV and PVB19). The median time to the onset of PVB19 disease in transplant recipients has been documented to be approximately 7 weeks after transplantation, like our case. Notably, among adult kidney transplant patients experiencing anemia, the incidence of positive PVB19 DNA rises significantly to approximately 27.4% [1].

Kidney transplant recipients, particularly those who received kidneys from donors after circulatory death, and those using immunosuppressant agents such as ATG, tacrolimus or MMF, may be at a higher risk of developing PVB19-related complications [2]. While some clinical guidelines for the management of PVB19 infection are available, such as those recommended by the American Society of Transplantation, no universal consensus exists in clinical practice regarding the ideal treatment for PVB19 infection. These guidelines typically suggest a reduction of immunosuppression at the time of diagnosis and IVIG administration. Therefore, management decisions should be made on a case-by-case basis, considering the unique clinical characteristics and needs of the patient [3].

Though the efficacy of IVIG as first line treatment of PVB19-related PRCA has been reported, no consensus exists on whether a low (0.25 g/kg) or high dose (2 g/kg) regimen is preferable. Some experts have suggested that a higher total dose of 2 g/kg of IVIG divided over 5 days could be useful to effectively eradicate the virus, particularly in patients with PVB19 infection after kidney transplantation [4]. This dose can be repeated if the patient’s infection relapses. In our case, IVIG was administered at a high dose but at longer intervals (four doses in a month, and monthly), demonstrating the lack of standardized treatment and the importance of tailoring treatment to the individual's needs.

A second-line approach in managing kidney transplant recipients with PVB19-related anemia involves modifying their immunosuppressive regimen. IVIG treatment in combination with the reduction of immunosuppressive medication has been reported as beneficial in cases of relapsing severe anemia due to PVB19 infection within the first 6 months after kidney transplantation [5].

An ongoing debate exists about the immunosuppressive agents, dose reduction, withdrawal, and shift to another medication. The introduction of mTOR inhibitors, particularly everolimus, provides a promising alternative in managing PVB19-related anemia. Everolimus inhibits cellular proliferation, offering distinct advantages in managing PVB19-related anemia. Its antiviral properties enhance the efficacy of memory T cells against viral cell growth [6]. In the present case two key immunosuppressive (IS) treatment adjustments were implemented: a delicate reduction in tacrolimus and MMF dosages was introduced and everolimus replaced mycophenolate mofetil (MMF).

In the case of recurrent and severe anemia in immunosuppressed pediatric patients, the possibility of PVB19 infection should be considered as well as a standardized approach and treatment options developed for cases involving PVB19 complications and sequelae. Guidelines on reducing immunosuppression and potential medication changes in such cases should be developed.

## Summary

### What is New?

PVB19 infection in pediatric kidney transplant recipients with severe and recurrent anemia require standardized approaches to treatment including immunosuppression and passive immunization.
